# Trends in treatment with antipsychotic medication in relation to national directives, in people with dementia – a review of the Swedish context

**DOI:** 10.1186/s12888-017-1409-9

**Published:** 2017-07-14

**Authors:** Staffan Karlsson, Ingalill Rahm Hallberg, Patrik Midlöv, Cecilia Fagerström

**Affiliations:** 1 Department of Health Sciences, Lund University, -221 00 Lund, SE Sweden; 2 School of Health and Welfare, Halmstad University, -301 18 Halmstad, SE Sweden; 3 Pufendorf Institute, Lund University, -221 00 Lund, SE Sweden; 4 Department of Clinical Sciences, Malmo, Lund University, -221 00 Lund, SE Sweden; 5 Blekinge Centre of Competence, -371 81 Karlskrona, SE Sweden; 6 Department of Health and Caring Sciences, Linnaeus University, -391 82 Kalmar, SE Sweden

**Keywords:** Dementia, Antipsychotic medication, Drug utilization, Directive

## Abstract

**Background:**

The aim of this study was to explore trends in treatment with antipsychotic medication in Swedish dementia care in nursing homes as reported in the most recent empirical studies on the topic, and to relate these trends to directives from the national authorities.

**Methods:**

The study included two scoping review studies based on searches of electronic databases as well as the Swedish directives in the field.

**Results:**

During the past decade, directives have been developed for antipsychotic medication in Sweden. These directives were generic at first, but have become increasingly specific and restrictive with time. The scoping review showed that treatment with antipsychotic drugs varied between 6% and 38%, and was higher in younger older persons and in those with moderate cognitive impairment and living in nursing homes for people with dementia. A decreasing trend in antipsychotic use has been seen over the last 15 years.

**Conclusions:**

Directives from the authorities in Sweden may have had an impact on treatment with antipsychotic medication for people with dementia. Treatment with antipsychotic medication has decreased, while treatment with combinations of psychotropic medications is common. National directives may possibly be even more effective, if applied in combination with systematic follow-ups.

**Electronic supplementary material:**

The online version of this article (doi:10.1186/s12888-017-1409-9) contains supplementary material, which is available to authorized users.

## Background

Dementia is on the increase and is having a _impact on health care around the world [1]. In Sweden about 5% of people 65 years and older receive public health care and social service in a nursing home [2], and of these about 70% [3] are known to have some degree of dementia. Clinical manifestations of dementia include a decline in memory and other cognitive functions as well as behavioural and psychological symptoms of dementia (BPSD) [4, 5]. Treatment options for BPSD have included antipsychotics [6, 7], although it is known that antipsychotic medication should be avoided particularly when treating people with a dementia disease because of the risk of side effects [8–10]. Despite the known risk, a European study comparing eight countries found high levels of antipsychotic medication in nursing homes in some of the countries, as well as great variation in treatment with antipsychotic medication. The lowest rate of antipsychotic treatment was found in the Swedish sample, where 11.9% of residents were given antipsychotics, as compared with Spain, where 50.4% received antipsychotic treatment. The other countries included also had a comparatively higher rate of antipsychotic use, ranging from 26.5% in France, 29.5% in Finland and 32.9% in the UK, to 35.4% in the Netherlands, 47.1% in Germany and 47.8% in Estonia [11]. The data were collected in 2012 and the explanation suggested for the lower rate in Sweden was that there have been national initiatives informing practice that antipsychotic medication should be avoided or used for only short periods. Such directives may have an impact on quality of care and services as well as on medical treatment of BPSD [12, 13].

Previous studies in Europe and the US have investigated the effect of national directives on the use of antipsychotic medication in persons with dementia and the temporal relation between the directives and the prescribing of these drugs. Their results showed that the impact of national directives on the use of antipsychotics varied across the studies [14–17]. The Swedish directives in 2000 concerning medical treatment in the elderly in general come from the National Board of Health and Social Welfare (NBHW) [18], among other sources. They emphasize the need to be especially careful when treating people with dementia. It may be that these directives have had an impact on practice and although it is hard to determine causal effects it seems worthwhile to explore any relationship between these national directives and practice.

The evidence on potential risks of psychotropic drugs, and particularly antipsychotic drugs, in dementia has been put forward, the risk for side effects of different kinds depending on whether these are first or second-generation drugs. There is evidence for the use of antipsychotics for specific symptoms such as BPSD or neuropsychiatric symptoms, although this effect is modest and the medication needs to be regularly reviewed and/or used cautiously because of risk of side effects. A review from 2005 concluded that pharmacological treatment in general was not particularly effective for management of neuropsychiatric symptoms of dementia [19]. Further, the review regarded the effect of antipsychotic medication as modest. Despite this finding, several studies in the review showed that antipsychotic medication in dementia care continues and is prescribed in particular for treatment of BPSD. A recent register study of older people (*n* = 641,566) showed that 4% of the sample used antipsychotic medication. Among persons diagnosed with dementia, 21% were treated with antipsychotics [20]. There was an increase from 11.6% to 12.8% in treatment with antipsychotics in those with dementia cared for at non-institutions. At the same time, there was a decrease in treatment with antipsychotics in those with dementia living in institutions, from 29.0% to 25.3%. No firm conclusions can be drawn about whether this represents a true increase or a decrease since these Swedish samples differ between the two points of assessment [21].

Antipsychotic medication has been commonly used in dementia treatment despite various side effects, which differ for different drug generations. Antipsychotics have been used even when the person’s behaviour does not suggest psychosis. As there is no clear evidence for treatment with antipsychotic medication among older people with dementia, a review of empirical studies and national directives may be beneficial.

## Aim

The aim of this study was to explore trends in treatment with antipsychotic medication in Swedish dementia care by reviewing empirical studies between 2000 and 2014 and relating the findings to directives from national authorities.

Specific objectives were to:

- Identify directives from the national authorities regarding treatment with antipsychotic drugs for older people, in particular people with dementia.
- Identify and explore empirical studies on antipsychotic drugs used in treating people with dementia living in care units, before and during the time when national directives concerning antipsychotic drug treatment were published.

## Methods

To answer the research questions, we included two scoping review studies. The scoping studies were reviews of Swedish directives as well as of published data in electronic databases in the field.

### Review of directives published by the Swedish authorities

In this study we have used the term “directive” as a synonym for “guideline”. The different authorities use various terminology, but we have chosen to use one term only. This study does not intend to evaluate the selected directives.

Targeted searches across the health care disciplines were performed regarding older people with dementia and received treatment with antipsychotics. Authorities whose directives were reviewed were the Swedish National Board of Health and Welfare (NBHW), the Swedish Council on Health Technology Assessment (SBU) and the Medical Products Agency (MPA). These are the governmental authorities that are eligible to issue health-related directives in Sweden. The search covered people aged 65+ receiving care at home or in a nursing home; treatment with medication including psycholeptics (anatomical therapeutic chemical (ATC) classification code N05) and psychoanaleptics (ATC code N06) [22]; and regulation of prescription. The ATC classification system is a standard for international drug utilization research. Published reports including directives before 15 November 2014 were searched for on the Internet home page of each authority. In addition, to validate the search and obtain a comprehensive review, an expert from each authority was contacted. The experts received the criteria for the search and then sent a list of the requested publications before 30 December 2014. Nine reports including directives regarding older people with dementia and treatment with antipsychotics were found. The reports were all published between 2004 and 2014 (Additional file 1).

### Scoping review of empirical studies

**Fig. 1:**
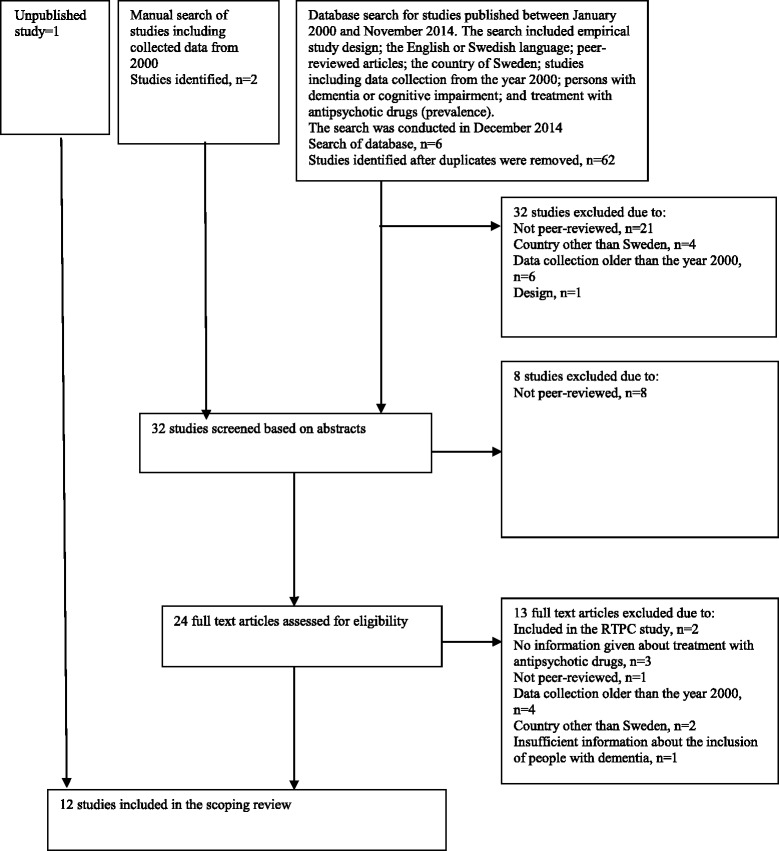
Flow diagram describing the process of selecting peer-reviewed studies based on the PRISMA statement (Moher, Liberati, Tetzlaff, Altman, & the PRISMA Group, 2009) [26] including the identification, screening, eligibility and inclusion phases

The review was conducted according to Arksey and O’Malley’s suggestion of a methodology for conducting scoping studies [23]. The systematic search was made in the electronic databases PubMed Central (PubMed), Medline databases (Medline), PsycINFO database (PsycINFO), Excerpta Medica dataBASE (Embase), the Cumulative Index to Nursing & Allied Health (Cinahl), and SveMed + database (SveMed+). The search strategy included terms, both from the indexes of the actual databases and from free text in each database, that were combined with “AND” and “OR” and were searched using truncation. A large number of terms or synonyms of “psychotropic agent” or “antipsychotic agent”, “dementia”, “institutional care” or “care”, “aged 65+” and “Sweden” or “Scandinavia” were applied [24]. Two of the authors screened the remaining search results from all levels of screening (i.e. titles, abstracts and full text reviews) using a pre-defined protocol. The screening of titles resulted in removal of 32 references due to: publication in non-peer-reviewed journals or practical journals (professional magazines), data collection having taken place before the year 2000, design issues, or non-inclusion of the Swedish context. After screening the abstracts, eight more references were removed and 13 references were read at full-text level, in accordance with the protocol (Fig. 1).

Reading the references in their entirety revealed that eleven references matched the inclusion criteria. The literature search was supplemented with a search of reference lists of relevant papers and resulted in no additional references. The results of the studies were read to determine the trend in antipsychotic treatment in older people with cognitive impairment or dementia living in care units (i.e. in a nursing home and elderly care unit) over the 15-year observation period (Additional file 2). Where additional information about treatment with antipsychotics was needed, we contacted the authors of the studies. The approach resulted in one additional unpublished study which was published in 2016, and unpublished data from two studies including older people living in elderly care units: The Swedish National Study on Aging and Care in Blekinge (SNAC-B) [25] and the RightTimePlaceCare (RTPC) study [26]. Some of the studies also revealed information about kind of antipsychotics used (conventional or atypical) and this information was used in the compilation.

## Results

### Directives from national authorities

Directives from national authorities were mainly based on evidence from research, such as the finding that antipsychotic medication treatment in BPSD has limited effect [27]. Further, it was stated that treatment with antipsychotic medication increases side effects such as risk of falls (odds ratio (OR) 1.28) and mortality (OR 2.32), and that simultaneous treatment with other psychotropics increases these risks [27–29]. During the last decade, several directives related to antipsychotic medication have been made, at first intending to explain the existing directives, and then making them more specific and restrictive. In 2004, the NBHW stated that the same physician should have responsibility for an older person’s medication in primary out-patient care and in long-term care, based on directives on follow-up and indicators for assessment of the medication [30]. The directives from the MPA in 2008 recommended that the physician in dementia care should consider ceasing antipsychotics treatment, or at least gradually decrease the dosage [31]. In 2010 the NBHW stated that in exceptional cases, medication with antipsychotics for psychosis and agitation may be considered when the person with dementia is suffering from delusion and hallucination [32]. On the whole, however, the NBHW stated that antipsychotic medication should be avoided because of considerable anticholinergic effects [28]. It is of particular importance to state indications for antipsychotic drug treatment and give correct and clear instructions when prescribing antipsychotics in older persons with dementia [28]. Further, the NBHW specified that a low dose of antipsychotic medication should be tested initially. The medication should be prescribed for a short treatment period only, with evaluation of the effect and possible side effects within 2 weeks. Furthermore, the number of persons with dementia in nursing homes, who are being treated with antipsychotic medication, or in units with established routines for following up treatment with antipsychotic medication, should be reported in accordance with directives from the county council or municipality [33, 34]. In 2013, directives from the NBHW pointed out that there is a need for a multidisciplinary approach to manage mental illness in older people, including those with dementia [29]. As antipsychotics are highly associated with a number of negative side effects, the directives from the NBHW became even more restrictive in 2014 regarding treatment in older people in medical health care and long-term care. Close monitoring of the medication is essential; a low dosage should be applied; treatment should be planned for a short time; there should be an early evaluation of effects and side effects; and treatment cessation and reduced dosage should be considered at frequent intervals [35] (Additional file 1).

### Prevalence of antipsychotics in the care of older people with dementia

The scoping review of the empirical study resulted in twelve studies [36–47] and unpublished data from two studies including older people. All studies included people with cognitive impairment or dementia diagnosis living in care units, nursing homes or geriatric settings, except for one study based on data taken from a national quality register [36]. Several studies (*n* = 9) were limited to a small part of Sweden, the county of Västerbotten and the surrounding communities located in northern Sweden. Eleven studies had a cross-sectional design and three had a longitudinal design. The prevalence of treatment with antipsychotic drugs differed between 6% and 38%. The use of antipsychotics was higher in younger persons, and in those with moderate cognitive impairment and those living in nursing homes for people with dementia. Some of the people with cognitive impairment were prescribed more than one psychotropic drug and most of them remained on drug treatment after 6 months [37, 38] (Additional file 2).

**Fig. 2:**
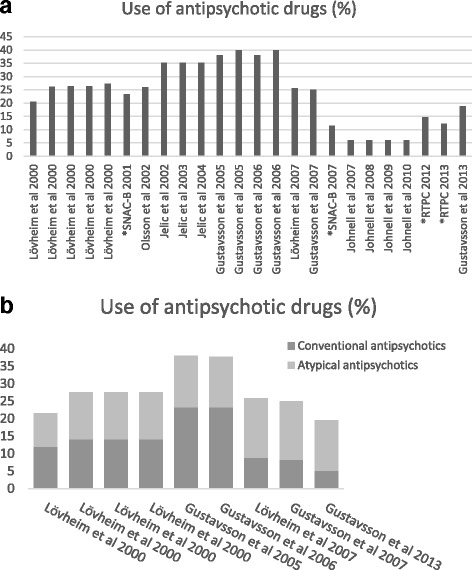
**a.** Included studies in the review. The histogram demonstrates the trend of use of antipsychotic drugs in Sweden from year 2000 to 2014. On the x-axel the authors and the data collection year is described. * represents unpublished data from the two studies SNAC-B and RTPC. **b.** In some of the studies included in the review the share of conventional and atypical antipsychotics was mentioned. The histogram demonstrates the trend of use of antipsychotic drugs in Sweden from year 2000 to 2014. On the x-axel the authors and the data collection year is described

Combinations of treatment with psycholeptic and psychoanaleptic drugs were found (42, 45, 47). In one overview, decreased prescribing of antipsychotics over time was identified (Fig. 2a), although the use of other psycholeptics and antidepressant drugs was unchanged over time in two of the reviewed studies (45, 47). Moreover, altered treatment trends concerning conventional and atypical antipsychotics were observed over time (Fig. 2b). The share of each type of drug changed over time, with atypical drug use increasing, from about half to two-thirds, and conventional drugs being prescribed less frequently.

## Discussion

The results show that the national directives regarding antipsychotic medication have become more specific and restrictive over time; simultaneously, antipsychotic treatment has appeared to decrease. The treatment with a combination of other psychotropic medications appears, however, to have remained unchanged and to be common.

With regard to national directives regarding antipsychotic medication becoming increasingly restrictive and specific over time, we found that around 2004, the directives recommended continuity in the prescribing physician and to consider revising the prescription of antipsychotics [30, 31]. Recent directives have advised that mental problems in older people should be treated in a multidisciplinary fashion, and that antipsychotic medication should be monitored and reported to the health authorities [29, 33–35]. In the present study, the restrictions contained in the directives may have decreased treatment with antipsychotic medication, which has also been found in previous studies [48, 49]. In 2004 the directives concerned regulations on continuity regarding the prescribing physician [30]. Previous studies have shown that a significant predictor of being prescribed inappropriate drugs was the presence of more than one prescriber [50, 51]. The most recent directives include reporting antipsychotic treatment to the authorities, and making this information available to the public. Earlier studies have found a slight effect of public reporting on care quality, but only in selected measures [52, 53]. It therefore appears that the strategies of increasing restrictiveness in directives may have improved the treatment with antipsychotic medication in dementia care in Sweden. Further studies about how directives are formulated and delivered from the prescriber’s view may improve the directives, making them more suitable and making their implementation even more efficient.

The findings of this study indicate that national directives may have had an impact on antipsychotic treatment during the 15-year observation period. A decrease in treatment with antipsychotics was observed, especially from 2005 to 2014. In 2005 about 40% of older people were prescribed antipsychotics, compared with about 20% in 2014. A previous study from Sweden, comparing data from 1987 to 1989 and 1994–1996, showed varying results, including increased use of antipsychotics among persons with dementia living at home, while antipsychotic treatment among persons in institutions decreased over the same period [21]. Similar developments have been shown in other countries. One study from the US reports that the legislation of the Federal Nursing Home Reform Act from the Omnibus Budget Reconciliation Act of 1987 (OBRA-87) coincided with a decrease in antipsychotic drug use in nursing home care [14]. Another study from the UK indicated that a National Dementia and Antipsychotic Prescribing Audit decreased prescriptions of antipsychotic medication for persons with dementia from about 17% in 2006 to about 7% in 2011 [15]. It appears that national directives have an impact on decreased antipsychotic use, which may imply improved care for persons with dementia.

Discussions among health professionals about directives (and preparatory work prior to the publication of directives) may in addition have had an impact on treatment trends. The directives were formulated in response to research findings that antipsychotic medication carries a risk for side effects such as falls and mortality [27–29]. The clinical trials involved may have had an impact in reducing treatment with antipsychotic medication. At the same time, studies were emphasizing non-pharmacological treatment, such as psychological and training interventions, for effectively reducing BPSD in people with dementia [54, 55]. Directives that focused specifically on antipsychotic medication were first established in 2008. These directives were very restrictive and emphasized treatment with clear indications, for a short period only and at low dosage; they further emphasized the importance of evaluating effects and side effects [28, 29, 32]. In addition, they recommended that prevalence and follow-up be reported to the authorities [33, 34]. In response to these directives, treatment with antipsychotics decreased even further and the latest studies showed a prevalence of 10–20% of antipsychotics use in older persons. Consequently, the results of this study may indicate a relation between national directives and treatment with antipsychotics in older persons. Although the decrease in antipsychotic use is consistent with the implementation of national directives, further research is needed to determine the effects on quality of nursing home care and outcomes in residents.

When directives regarding antipsychotic use became more restrictive other psychotropic medication was introduced. An increased awareness among the health professionals of negative side effects of antipsychotics may have resulted in treatment with other medications. The findings indicate that the share of each antipsychotic drug type changed over time, i.e. atypical drug use increased, from about half to two-thirds, and conventional drugs were prescribed less frequently. Previous studies have reported that other psychotropic medication has substituted antipsychotics in the management of neuropsychiatric symptoms [56, 57]. However, other studies have shown only a small, or no, increase use of other psychotropic medication, while the use of antipsychotics has decreased [14, 15]. The replacing of antipsychotic use with other psychotropic medication appears to vary in proportion. Further research about the use of psychotropic medication replacing antipsychotics and about non-pharmacological interventions, including their effectiveness, is essential.

## Methodological considerations

The focus in this study was on identifying trends of prescribing antipsychotic drugs in the care of older people, particularly older people with dementia, in Sweden. We aimed to explore how national directives have impacted treatment with antipsychotic medication during the last decade. To find out the directives’ impact on utilization, we used two scoping reviews. The content of the directives was analysed, but how and to which extent they were implemented in practice is unknown. This means that the interventions made in response to the presented directives are not known, which can be considered a weakness. Consequently, this study has not succeeded in establishing causal relations between the national directives and treatment with antipsychotic medication. Moreover, other interventions besides national directives may have had an impact on treatment with antipsychotic medication during the study period.

In the present study, data were collected in the two scoping reviews, based on scientific papers as well as on directives from public authorities. It may be a strength that the data were drawn from two sources related to the aim. In the scoping review, nine out of twelve studies were conducted in the same geographic area in Sweden and the data were drawn from similar samples. The results may therefore have limited generalizability to the rest of Sweden. However, the results from the scoping review were strengthened by the unpublished empirical studies using samples from other parts of the country.

## Conclusions

The restrictions in directives from the Swedish national authorities during the 15-year observation period seem to have had an impact on antipsychotic drug medication in people with dementia. The results show that treatment with antipsychotic medication has decreased, a positive trend since directives recommend that antipsychotic drugs should be avoided because of negative side effects in old age. Still, treatment with combinations of other psychotropic medications appears to remain unchanged and to be common. In the development of new national directives, a supplement to the directives, detailing systematic follow-ups, will perhaps make the directives even more effective.

## Additional files

Additional file 1: Public authorities’ recommendations regarding psychotropic utilization in older persons with dementia. BPSD = behavioural and psychological symptoms of dementia; MPA = Medical Products Agency; NBHW = National Board of Health and Welfare; OR = odds ratio; SALAR = Swedish Association of Local Authorities and Regions; SBU = the Swedish Council on Health Technology Assessment; SveDem = the Swedish Dementia Registry; SSRI = selective serotonin reuptake inhibitor. (DOCX 31 kb)

Additional file 2: Overview of the studies included in the scoping review*.* ADL = Activities of Daily Living; BPSD = Behavioural and Psychological Symptoms of Dementia; MDDAS = Multi-Dimensional Dementia Assessment Scale; MMSE = Mini Mental State Examination; SIB = Severe Impairment Battery; ADCS-ADL = Alzheimer’s disease Cooperative Study-activities of daily living scale; NPI = Neuro-psychiatric Inventory; CGI-I = Clinical Global Impression of Improvement. SNAC-B = The Swedish National Study of Ageing and Care in Blekinge, RTPC = RightTimePlaceCare project. (DOCX 27 kb)

## Abbreviations

ATC: anatomical therapeutic chemical
BPSD: behavioural and psychological symptoms of dementia
Cinahl: Cumulative Index to Nursing & Allied Health.
Embase: Excerpta Medica dataBASE
MPA: Medical Products Agency
n: number
NBHW: National Board of Health and Welfare
NPI-Q: Neuropsychiatric Inventory Questionnaire
OBRA-87: Omnibus Budget Reconciliation Act of 1987.
OR: odds ratio
p: *p*-value
RTPC: RightTimePlaceCare
SBU: Swedish Council on Health Technology Assessment
SNAC-B: Swedish National Study on Aging and Care-Blekinge

## Abbreviations that appear in the supplementary material only

ADCS**–**ADL: Alzheimer’s Disease Cooperative Study**–**activities of daily living
CGI-I: clinical global impression of improvement
MDDAS: Multi-Dimensional Dementia Assessment Scale
SALAR: Swedish Association of Local Authorities and Regions
SIB: Severe Impairment Battery
SSRI: selective serotonin reuptake inhibitor
SveDem: The Swedish Dementia Registry

## References

[1] Ferri CP, Prince M, Brayne C, Brodaty H, Fratiglioni L, Ganguli M, Hasegawa K, Hendrie H, Huang Y, Jorm A, Mathers C, Menezes PR, Rimmer E, Scazufca M (2005). Global prevalence of dementia: a Delphi consensus study. Lancet.

[2] The National Board of Health and Welfare: Äldre och personer med funktionsnedsättning– regiform år 2014 Elderly and persons with impairments- management form, 2014. Some municipal services according to the Social Services Act. Stockholm: The National Board of Health and Welfare; 2015.

[3] The National Board of Health and Welfare: Nationell utvärdering – Vård och omsorg vid demenssjukdom 2014 – Indikatorer och underlag för bedömningar (National evaluation – Care in cases of dementia – Indicators and basis for assessment). Stockholm: The National Board of Health and Welfare; 2014.

[4] Fauth EB, Gibbons A (2014). Which behavioural and psychological symptoms of dementia are the most problematic? Variability by prevalence, intensity, distress ratings, and associations with caregiver depressive symptoms. Int J Geriatr Psychiatry.

[5] Linde RM, Stephan B, Matthews FE, Brayne C, Savva GM (2013). The presence of behavioral and psychological symptoms and progression to dementia in the cognitively impaired older population. Int J Geriatr Psychiatry.

[6] Sultzer DL (2004). Psychosis and antipsychotic medications in Alzheimer's disease: clinical management and research perspectives. Dement Geriatr Cogn Disord.

[7] Rochon PA, Stukel TA, Bronskill SE, Gomes T, Sykora K, Wodchis WP, Hillmer M, Kopp A, Gurwitz JH, Anderson GM (2007). Variation in nursing home antipsychotic prescribing rates. Arch Intern Med.

[8] Ballard C, Lana MM, Theodoulou M, Douglas S, McShane R, Jacoby R, Kossakowski K, Yu LM (2008). Juszczak E, on behalf of the Investigators DART AD: A randomised, blinded, placebo-controlled trial in dementia patients continuing or stopping neuroleptics (the DART-AD trial). PLoS Med.

[9] Mittal V, Kurup L, Williamson D, Muralee S, Tampi RR (2011). Risk of cerebrovascular adverse events and death in elderly patients with dementia when treated with antipsychotic medications: a literature review of evidence. Am. J. Alzheimers Dis. Other Dement.

[10] Oderda LH, Young JR, Asche CV, Pepper GA (2012). Psychotropic-related hip fractures: meta-analysis of first-generation and second-generation antidepressant and antipsychotic drugs. Ann. Pharmacother.

[11] de Mauleon A, Sourdet S, Renom-Guiteras A, Gillette-Guyonnet S, Leino-Kilpi H, Karlsson S, Bleijlevens M, Zabategui A, Saks K, Vellas B, Jolley D, Soto M (2014). Associated factors with antipsychotic use in long-term institutional care in eight European countries: Results from the RightTimePlaceCare study. J Am Med Dir Assoc.

[12] Mukamel DB, Weimer DL, Harrington C, Spector WD, Ladd H, Li Y (2012). The effect of state regulatory stringency on nursing home quality. Health Serv Res.

[13] Bowblis JR, Lucas JA (2012). The impact of state regulations on nursing home care Practices. J Regul Econ.

[14] Shorr RI, Fought RL, Ray WA (1994). Changes in antipsychotic drug use in nursing homes during implementation of the OBRA-87 regulations. JAMA.

[15] National Health Service (UK): National Dementia and Antipsychotic Prescribing Audit 2012. Leeds: National Health Service; 2012.

[16] Desai VC, Heaton PC, Kelton CM (2012). Impact of the Food and Drug Administration's antipsychotic black box warning on psychotropic drug prescribing in elderly patients with dementia in outpatient and office-based settings. Alzheimer's & Dementia.

[17] Gallini A, Andrieu S, Donohue JM, Oumouhou N, Lapeyre-Mestre M, Gardette V (2014). Trends in use of antipsychotics in elderly patients with dementia: Impact of national safety warnings. European Neuropsychopharmacology.

[18] The National Board of Health and Welfare: Kvaliteten på läkemedelsanvändningen hos äldre (The quality of medication utilization in older people). Stockholm: The National Board of Health and Welfare; 2000.

[19] Sink KM, Holden KF, Yaffe K (2005). Pharmacological treatment of neuropsychiatric symptoms of dementia: a review of the evidence. JAMA.

[20] Giron MS, Forsell Y, Bernsten C, Thorslund M, Winblad B, Fastbom J (2001). Psychotropic drug use in elderly people with and without dementia. Int J Geriatr Psychiatry.

[21] Wastesson JW, Ringbäck WG, Johnell K (2015). Educational disparities in antipsychotic drug use among older people with and without dementia in Sweden. Acta Psychiatr Scand.

[22] WHO Collaborating Centre for Drug Statistics Methodology (2014). Guidelines for ATC classification and DDD assignment 2015.

[23] Arksey H, O’Malley L (2005). Scoping Studies: Towards a Methodological Framework. Int. J. Social Research Methodology.

[24] Moher D, Liberati A, Tetzlaff J, Altman DG, PRISMA Group: Preferred reporting items for systematic reviews and meta-analyses: the PRISMA statement. Int J Surg 2009, 8:336–341. PMC3090117 21603045

[25] Lagergren M, Fratiglioni L, Hallberg IR, Berglund J, Elmståhl S, Hagberg B, Holst G, Rennemark M, Sjölund BM, Thorslund M, Wiberg I, Winblad B, Wimo A: A longitudinal study integrating population, care and social services data. The Swedish National Study on Aging and Care (SNAC). Aging Clin Exp Res 2004, 16:158–168. 10.1007/BF03324546 15195992

[26] Verbeek H, Meyer G, Leino-Kilpi H, Zabalegui A, Hallberg IR, Saks K, Soto ME, Challis D, Sauerland D, Hamers JP (2012). A European study investigating patterns of transition from home care towards institutional dementia care: the protocol of the RightTimePlaceCare study. BMC Public Health.

[27] The Swedish Council on Health Technology Assessment (SBU): Demenssjukdomar, En systematisk litteraturöversikt (Dementia diseases. A systematic literature review). Stockholm: SBU; 2006.

[28] The National Board of Health and Welfare (NBHW) a: Indikatorer för god läkemedelsterapi hos äldre (Indicators for good medication among older people). Stockholm: NBHW; 2010.

[29] The National Board of Health and Welfare (NBHW): Psykisk sjukdom bland äldre och behandling inom vården (Mental illness among elderly and treatment within health care). Stockholm: NBHW; 2013.

[30] The National Board of Health and Welfare (NBHW): Uppföljning av äldres läkemedels-användning (Follow up of older peoples utilization of medication). Stockholm: NBHW; 2004.

[31] Medical Products Agency (MPA): Läkemedels-behandling och bemötande vid beteendemässiga och psykiska symtom vid demenssjukdom-BPSD (Medication treatment and encountering behaviour and psychological symptoms in dementia disease-BPSD). Stockholm: MPA; 2008.

[32] The National Board of Health and Welfare (NBHW) b: Nationella riktlinjer för vård och omsorg vid demenssjukdom 2010. (National guidelines for care and service in dementia disease). Stockholm: NBHW; 2010.

[33] The National Board of Health and Welfare (NBHW) c: Indikatorer. Nationella riktlinjer för vård och omsorg vid demenssjukdom 2010. Indikator 3. Bilaga 3 (Indicators. National guidelines for care and service in dementia disease 2010. Indicator 3. Appendix 3). Stockholm: NBHW; 2010.

[34] The National Board of Health and Welfare (NBHW) d: Indikatorer. Nationella riktlinjer för vård och omsorg vid demenssjukdom 2010. Indikator 2. Bilaga 3 (Indicators. National guidelines for care and service in dementia disease 2010. Indicator 2. Appendix 3). Stockholm: NBHW; 2010.

[35] The National Board of Health and Welfare (NBHW): Öppna jämförelser 2014 – Läkemedels-behandlingar – Jämförelser mellan landsting. (Open comparisons 2014. Medication treatment. Comparisons between county councils). Stockholm: NBHW; 2014.

[36] Johnell K, Religa D, Eriksdotter M (2013). Differences in drug therapy between dementia disorders in the Swedish dementia registry: A nationwide study of over 7,000 patients. See comment in PubMed Commons belowDement Geriatr Cogn Disord.

[37] Gustavsson M, Karlsson S, Lovheim H (2013). Inappropriate long-term use of antipsychotic drugs is common among people with dementia living in specialized care units. BMC Pharmacol Toxicol.

[38] Gustavsson M, Karlsson S, Gustavsson Y, Lovheim H (2013). Psychotropic drug use among people with dementia – a six-month follow-up study. BMC Pharmacol Toxicol.

[39] Lovheim H, Sandman P-O, Kallin K, Karlsson S, Gustafson Y (2008). Symptoms of mental health and psychotropic drug use among old people in geriatric care, changes between 1982 and 2000. Int J Geriatr Psychiatry.

[40] Lövheim H, Sandman PO, Karlsson S, Gustafson Y (2009). Changes between 1982 and 2000 in the prevalence of behavioral symptoms and psychotropic drug treatment among old people with cognitive impairment in geriatric care. Int Psychogeriatr.

[41] Lovheim H, Sandman P-O, Kallin K, Karlsson S, Gustavsson Y (2006). Relationship between antipsychotic drug use and behavioural and psychological symptoms of dementia in old people with cognitive impairment living in geriatric care. Int Psychogeriatr.

[42] Lövheim H, Bergdahl E, Sandman PO, Karlsson S, Gustafson Y (2010). One-week prevalence of depressive symptoms and psychotropic drug treatments among old people with different levels of cognitive impairment living in institutional care: changes between 1982 and 2000. Int Psychogeriatr.

[43] Olsson J, Bergman A, Carlsten A, Oké T, Bernsten C, Schmidt IK, Fastbom J (2010). Quality of drug prescribing in elderly people in nursing homes and special care units for dementia: a cross-sectional computerized pharmacy register analysis. Clin Drug Investig.

[44] Jelic V, Haglund A, Kowalski J, Langworth S, Winblad B (2008). Donepezil treatment of severe Alzheimer's disease in nursing home settings. A responder analysis. Dement Geriatr Cogn Disord.

[45] Lövheim H, Gustafson Y, Karlsson S, Sandman PO (2011). Comparison of behavioral and psychological symptoms of dementia and psychotropic drug treatments among old people in geriatric care in 2000 and 2007. Int Psychogeriatr.

[46] Gustafsson M, Sandman PO, Karlsson S, Gustafson Y, Lövheim H (2013). Association between behavioral and psychological symptoms and psychotropic drug use among old people with cognitive impairment living in geriatric care settings. Int Psychogeriatr.

[47] Gustavsson M, Isaksson U, Karlsson S, Sandman PO, Lövheim H. Behavioral and psychological symptoms and psychotropic drugs among people with cognitive impairment in nursing homes in 2007 and 2013. Eur J Clin Pharmacol. 2016, Apr;13 [Epub ahead of print] 10.1007/s00228-016-2058-5 27071994

[48] Bowblis JR, Crystal S, Intrator O (2012). Lucas, JA: Response to regulatory stringency: The case of antipsychotic medication use in nursing homes. Health Econ.

[49] Kales HC, Zivin K, Kim HM, Valenstein M, Chiang C, Ignacio RV, Ganoczy D, Cunningham F, Schneider LS, Blow FC (2011). Trends in antipsychotic use in dementia 1999–2007. Arch Gen Psychiatry.

[50] Olsson J, Bergman A, Carlsten A (2010). Oke´ T, Bernsten C, Schmidt IK, Fastbom J: Quality of Drug Prescribing in Elderly People in Nursing Homes and Special Care Units for Dementia. A Cross-Sectional Computerized Pharmacy Register Analysis. Clin Drug Investig.

[51] Dhalla IA, Anderson GM, Mamdani MM, Bronskill SE, Sykora K, Rochon PA (2002). Inappropriate prescribing before and after nursing home admission. J Am Geriatr Soc.

[52] Grabowski DC, Town RJ: Does information matter? Competition, quality, and the impact of nursing home report cards. Health Serv Res 2011, 46(6pt1):1698–1719. 10.1111/j.1475-6773.2011.01298.x PMC3393022 21790590

[53] Werner RM, Konetzka RT, Kruse GB (2009). Impact of public reporting on unreported quality of care. Health Serv Res.

[54] Livingston G, Kelly L, Lewis-Holmes E, Baio G, Morris S, Patel N, Omar RZ, Katona C, Cooper C (2014). A systematic review of the clinical effectiveness and cost-effectiveness of sensory, psychological and behavioral interventions for managing agitation in older adults with dementia. Health Technol Assess.

[55] Deudon A, Maubourguet N, Gervais X, Leone E, Brocker P, Carcaillon L, Riff S, Lavallart B, Robert PH (2009). Non-pharmacological management of behavioral symptoms in nursing homes. Int J Geriatr Psychiatry.

[56] Stephenson CP, Karanges E, McGregor IS (2013). Trends in the utilisation of psychotropic medications in Australia from 2000 to 2011. Aust N Z J Psychiatry.

[57] Vasudev A, Shariff SZ, Liu K, Burhan AM, Herrmann N, Leonard S, Mamdani M (2015). Trends in Psychotropic Dispensing Among Older Adults with Dementia Living in Long-Term Care Facilities: 2004–2013. Am J Geriatr Psychiatry.

