# Supplementary material for: Trends in treatment with antipsychotic medication in relation to national directives, in people with dementia – a review of the Swedish context
Source: BMC Psychiatry. 2017 Jul 14;17:251. doi: 10.1186/s12888-017-1409-9 (PMC5513361; doi:10.1186/s12888-017-1409-9)
Supplement: Supplementary file 1 — Public authorities’ recommendations regarding psychotropic utilization in older persons with dementia. BPSD = behavioural and psychological symptoms of dementia; MPA = Medical Products Agency; NBHW = National Board of Health and Welfare; OR = odds ratio; SALAR = Swedish Association of Local Authorities and Regions; SBU = the Swedish Council on Health Technology Assessment; SveDem = the Swedish Dementia Registry; SSRI = selective serotonin reuptake inhibitor. (DOCX 31 kb) [file 12888_2017_1409_MOESM1_ESM.docx]

**Public authorities’ recommendations regarding psychotropic utilization in older persons with dementia**

| No. | Authority/  year | | Title | Aim | Methodology/data sources | Findings/recommendations |
| --- | --- | --- | --- | --- | --- | --- |
| 1  [40] | The National Board of Health and Welfare, 2004 | Uppföljning av äldres läkemedels-användning. (Follow-up of older people’s utilization of medication.) | | To follow up and evaluate the utilization of medication in the elderly. | Review of three studies regarding utilization of medication. Survey of: managers in primary out-patient care, the responsible RNs, and chairmen of pharmaceutical committees. | Findings: The survey showed that the quality indicators for following up and reconsidering medication treatment of older people are well known. Follow-up of older people’s utilization of medication is lacking; improvements are needed. Physician continuity is a requirement for a satisfactory follow-up. The pharmaceutical committees should stake more on patient safety aspects. It seems to be unclear who is responsible to decide about the follow-up of older people’s utilization of medication.  Recommendations: The same physician should have the responsibility for the older person’s medication. There should be regulation in follow-up of older people’s medication. Indicators for assessment of older people’s medication should be part of the care activities. Education and in-service training of physicians and other health care staff is needed, as is a joint medication list and computerized support for prescribing. |
| 2  [37] | SBU, 2006 | Demenssjukdomar, En systematisk litteraturöversikt.  (Dementia diseases. A systematic literature review.) | | To establish the state of knowledge regarding medication treatment in dementia disease. | Systematic review of the scientific literature. | Findings: Medication treatment in BPSD has a limited effect. Increased mortality has been found when using atypical antipsychotic medication in treatment of BPSD in dementia. The effect has not been seen in individual studies, but has been establish in a meta-analysis. |
| 3  [41] | MPA, 2008 | Läkemedels-behandling och bemötande vid beteendemässiga och psykiska symtom vid demenssjukdom-BPSD. (Medication treatment and encountering behaviour and psychological symptoms in dementia disease-BPSD.) | |  |  | Recommendations:  1. Investigate/map symptoms, possible causes and triggering factors, and possibly also the burden of mental or physical diseases.  2. Review the medical treatment.  3. Optimize the caring environment and treatment.  4. Where effects are insufficient, a decision needs to be made about medical treatment:  - In depressive symptoms, SSRI medication is the first choice.  - In irritability, agitation and anxiety, SSRI medication may be tested.  - Memantine may have an effect primarily on agitation and aggressiveness.  - In psychotic symptoms and aggressiveness causing suffering for the patient and/or potential danger for the patient or others, a low dose of antipsychotic medication may be tested. The medication should be used very restrictively.  - Where acute sedation is needed, anxiolytics (N05B) may be tested for a short period and with adequate supervision.  - Where antipsychotics or anxiolytics are prescribed, this should be for a short period, with evaluation of effects and side effects within 2 weeks. Treatment cessation and/or a decrease in dosage should be considered regularly. |
| 4  [38] | The National Board of Health and Welfare, 2010 | Indikatorer för god läkemedelsterapi hos äldre. (Indicators for good medication among older people.) | | To update and develop indicators (previously suggested by the NBHW) for measuring and following quality in older people’s medication. | A workgroup within the NBHW made a recommendation on quality indicators for utilization of medication, assessed by an expert group and sent for review and consideration. This is a revision of the first version. | Recommendations included the following:  Quality indicators for medication may be applied: as support in prescribing medication to older people, in follow-ups and interventions (medication review), in follow-up of patterns in prescribing of medication, in national follow-ups and epidemiological studies.  Indicators are divided into medication-specific and diagnosis-specific. Indicators reflect utilization in a group, rather than individual cases.  Medication-specific indicators: benzodiazepine (N05B, N05C), which has a long half-life, medication with considerable anticholinergic effects (N05A, N05B, N06A) and propiomazine (N05C) should be avoided.  Including correct and clear indications with medicine is of particular importance (antipsychotics, N05A; antidepressants and SSRIs, N06A).  Inappropriate administration with risk for side effects or ineffective treatment (hypnotics, N05C; antipsychotics, N05A).  Inappropriate dosage of antipsychotics, N05A; anxiolytics, N05B; hypnotics, N05C, with risk of side effects.  Joint treatment with three or more psychotropic drugs (N05A, N05B, N05C, N06A) implies an increased risk for side effects and interactions.  Recommendations also included:  Medication that causes or aggravates a fall in blood pressure (N05A, N06A).  Medication that increases the risk for fall (N05A, N05B, N05C, N06A).  Medication that may cause cognitive disturbance (N05A, N05B, N05C).  Inappropriate and risky utilization of medication in persons with dementia disease. |
| 5  [44] | The National Board of Health and Welfare, 2010 | Indikatorer  Nationella riktlinjer för vård och omsorg vid demenssjukdom 2010. Indikator 2. Bilaga 3. (Indicators. National guidelines for care and service in dementia disease 2010. Indicator 2. Appendix 3.) | | Indicator 2. Treatment with antipsychotic medication in nursing homes. | Registers:  SveDem;  The Pharmaceutical Register;  The Elderly- and handicap service register. | Recommendations concerned the proportion of persons with dementia in nursing homes treated with antipsychotic medication; the yearly report at county council and municipal level, divided by gender and age. |
| 6  [43] | The National Board of Health and Welfare, 2010 | Indikatorer  Nationella riktlinjer för vård och omsorg vid demenssjukdom 2010. Indikator 3. Bilaga 3. (Indicators. National guidelines for care and service in dementia disease 2010. Indicator 3. Appendix 3.) | | Indicator 3.  Guidelines and routines for pharmaceutical survey with focus on follow-up of treatment with antipsychotic medication in persons with dementia. | Questionnaires to managers in primary health care and specialized health care. | Recommendations concerned the proportion of units with established routines for how treatment with antipsychotic medication is followed up, in relation to investigated units; and yearly report at county council and municipal level. |
| 7  [42] | The National Board of Health and Welfare, 2010 | Nationella riktlinjer för vård och omsorg vid demenssjukdom 2010. (National guidelines for care and service in dementia disease, 2010.) | | To provide knowledge-based recommendations concerning how municipalities, county councils, and private and voluntary providers should meet the needs for care and service of persons with dementia and their next of kin. | Literature review.  Statements from experts within dementia care. | Recommendations: In exceptional cases, when person-centred care and adaptation of the caring environment have been found insufficient and the condition causes suffering for the person with dementia, medication may be considered, using: Memantine for persons with Alzheimer’s disease and BPSD symptoms.  Benzodiazepine as short-term medication for anxiety.  Antipsychotics for psychosis and agitation.  Clomethiazole, to be taken at night, for dementia disease and BPSD, mental symptoms and confusion.  Antipsychotics may be used when the person is suffering from delusion and hallucination in moderate to severe Lewy body and Parkinson’s dementia. |
| 8  [39] | The National Board of Health and Welfare, 2013 | Psykisk sjukdom bland äldre och behandling inom vården. (Mental illness among elderly persons and treatment within health care.) | | To increase the knowledge concerning the characteristics of elderly people with mental illness and the health care they receive. | Data from the National Board of Health and Welfare national register; Patient register, Pharmaceutical register; and Cause of Death register. | Findings: The majority of prescriptions of psychotropics for older people, particularly the very old, were provided in primary health care. Treatment with antipsychotic medication in people aged 65+ increased the risks for side effects such as falls (OR 1.28) and mortality (OR 2.32). Simultaneous treatment with various psychotropics increased this risk even further. Older people with mental illness are treated unfairly and this may have serious consequences for the wellbeing of older people. There is a need to use a multidisciplinary approach to tackle mental illness in older people, as both physical and mental illness are present and coexist in older people. |
| 9  [45] | The National Board of Health and Welfare, 2014 | Öppna jämförelser 2014 – Läkemedels-behandlingar – Jämförelser mellan landsting. (Open comparisons, 2014. Medication treatment. Comparisons between county councils.) | | Open comparisons aim to stimulate the development of equal and effective medical health care with good quality. | Statistics on publicly financed medical health care in county councils and municipalities; data available nationally. | Findings and recommendations: In line with NBHW indicators for treatment with medication, zopiclone is the most appropriate hypnotic for older people, because of the relatively short-term effect.  Medications that should be avoided are benzodiazepine, because of its long half-life and considerable anti-cholinergic effects, and the hypnotic propiomazine. These should only be utilized in cases with well-founded and present indication, and when the predicted benefit is reasonably related to the risk. The treatment should be followed up and reconsidered at frequent intervals.  As antipsychotics are highly associated with a number of negative side effects, low dosage should be applied and a short treatment time planned, with early evaluation of effects and side effects. In addition, the treatment should be reconsidered at frequent intervals with a view to cessation or reduction of dosage. Furthermore, benzodiazepines with long-term effects are included in the indicator “Older people with medication that should be avoided”. |

BPSD = behavioural and psychological symptoms of dementia; MPA = Medical Products Agency; NBHW = National Board of Health and Welfare; OR = odds ratio; SALAR = Swedish Association of Local Authorities and Regions; SBU = the Swedish Council on Health Technology Assessment; SveDem = the Swedish Dementia Registry; SSRI = selective serotonin reuptake inhibitor.
