# Supplementary material for: Trends in treatment with antipsychotic medication in relation to national directives, in people with dementia – a review of the Swedish context
Source: BMC Psychiatry. 2017 Jul 14;17:251. doi: 10.1186/s12888-017-1409-9 (PMC5513361; doi:10.1186/s12888-017-1409-9)
Supplement: Supplementary file 2 — Overview of the studies included in the scoping review. ADL = Activities of Daily Living; BPSD = Behavioural and Psychological Symptoms of Dementia; MDDAS = Multi-Dimensional Dementia Assessment Scale; MMSE = Mini Mental State Examination; SIB = Severe Impairment Battery; ADCS-ADL = Alzheimer’s disease Cooperative Study-activities of daily living scale; NPI = Neuro-psychiatric Inventory; CGI-I = Clinical Global Impression of Improvement. SNAC-B = The Swedish National Study of Ageing and Care in Blekinge, RTPC = RightTimePlaceCare project. (DOCX 27 kb) [file 12888_2017_1409_MOESM2_ESM.docx]

**Overview of the studies included in the scoping review**

| Author(s), year | Title | Study population | Aim | Methodology | Outcome measures | Important results |
| --- | --- | --- | --- | --- | --- | --- |
| Lövheim H, Sandman P-O, Kallin K, Karlsson S, Gustafson Y, 2008 [41] | Symptoms of mental health and psychotropic drug use among old people in geriatric care, changes between 1982 and 2000. | N=6 864 person (aged ≥65 years) living in geriatric care units, in Västerbotten, Sweden.  Data collected in 1982 (n=3 195) and 2000 (n=3 669).  Mean age in 2000 was 83.3±7.0 years. | To compare the occurrence of certain symptoms and behaviours and the prevalence of psychotropic drug use in two cross-sectional surveys from 1982 and 2000, in a population consisting of all persons living in geriatric care units, aged ≥65 years, in the county of Västerbotten in northern Sweden. The results were controlled for demographical changes. | Cross-sectional at two occasions | Prescriptions were collected from the records; BPSD, ADL and mobility were collected using the MDDAS and observations by care staff. | In 2000 the prevalence of antipsychotics was 752/3 669 (20.5%), a decrease in comparison with 1982.  The prevalence of conventional antipsychotic use was 439/3 669 (12.0%) and of atypical antipsychotics 349/3 669 (9.5%). Conventional antipsychotics had decreased and atypical antipsychotics had increased between 1982 and 2000. |
| [Lövheim H](http://www.refworks.com/refworks2/default.aspx?r=references\|MainLayout::init), Sandman P-O, Karlsson S, Gustafson Y, 2009 [42] | Changes between 1982 and 2000 in the prevalence of behavioural symptoms and psychotropic drug treatment among old people with cognitive impairment in geriatric care. | N=3 404 persons (aged ≥65 years) with cognitive impairment living in geriatric care units, in Västerbotten, Sweden.  Data were collected in 1982 and 2000.  Mean age was 83.4±7.0 years in 2000. | To analyse and compare the 1-week prevalence of various behavioural symptoms and psychotropic drug treatment among people with cognitive impairment living in institutional care in two large, comparable samples from 1982 and 2000. | Cross-sectional at two occasions | Age, living arrangement and behavioural symptoms were collected using the MDDAS; cognitive impairment was assessed by the Gottfries’ cognitive scale; medical drugs and comorbidity were registered. | The prevalence of antipsychotic drug use decreased from 38.0% to 26.2% from 1982 to 2000. One-week prevalence of regressive symptoms and resistance to care had decreased.  Atypical antipsychotics, which were not available in 1982, accounted for about half of the prescriptions of antipsychotic drugs in 2000. |
| Lövheim H, Sandman P-O, Kallin K, Karlsson S, Gustafson Y, 2006 [43] | Relationship between antipsychotic drug use and behavioural and psychological symptoms of dementia in old people with cognitive impairment living in geriatric care. | N=2 017 residents aged ≥65 years (mean age 83.5 years) with cognitive impairment living in geriatric care units, in Västerbotten, Sweden.  Data were collected in 2000. | To discover factors associated with the use of antipsychotics. | Cross-sectional | Prescriptions were collected from the records, and symptoms/behavior, ADL and mobility were collected through observations made by care staff using the BPSD and MDDAS | Antipsychotics were used by 531 (26.3%), 285 (14.1%) of whom used conventional antipsychotics and 270 (13.4%) used atypical antipsychotics. |
| Lövheim H, Bergdahl E, Sandman P-O, Karlsson S, Gustafson Y, 2010 [44] | One-week prevalence of depressive symptoms and psychotropic drug treatments among old people with different levels of cognitive impairment living in institutional care: changes between 1982 and 2000. | N=6 864 persons aged ≥65 years with different levels of cognitive impairment living in geriatric care units, in Västerbotten, Sweden.  Data were collected in 1982 and 2000.  Mean age was 83.5±6.8 years in 2000. | To investigate the prevalence of depressive symptoms and of psychotropic drug treatment among old people with different severity of cognitive impairment, in two large, comparable, cross-sectional samples from 1982 and 2000. | Cross-sectional at two occasions | Age, living arrangements and behavioural symptoms were collected using the MDDAS; cognitive impairment was collected using the Gottfries’ cognitive scale; medical drugs and comorbidity were registered. | In 2000, 26.3% of people with cognitive impairment used antipsychotics. Further, 27.3% among those with severe cognitive impairment, 30.4% among those with moderate cognitive impairment, 21.0% of those with mild cognitive impairment and 14.3% in the no cognitive impairment group used antipsychotics. Antipsychotic drug use was less common in all groups with cognitive impairment, except the group with a cognitive score of ≥24, i.e. with no cognitive impairment compared with baseline.  Treatment with a combination of antidepressants and antipsychotics was uncommon in 1982 (51/3 195; 1.6%), while in 2000 this was more common (severe cognitive impairment: 67/660; 10.1%; moderate cognitive impairment: 113/642; 17.6%; mild cognitive impairment: 61/666; 9.2%; no cognitive impairment: 89/1 242 aged ≥65 years; 7.2%). |
| Olsson J, Bergman A, Carlsten A, Oké T, Bernsten C, Schmidt IK, Fastbom J, 2010 [45] | Quality of drug prescribing in elderly people in nursing homes and special care units for dementia: a cross-sectional computerized pharmacy register analysis | N=3 705 persons (aged ≥65 years) living in nursing homes and care units for dementia, in  Jönköping, Sweden. Data were collected in 2002.  Mean age in 2002 was 85 years. | To assess the extent and quality of drug prescribing in younger and older elderly residents receiving multi-dose drugs in ordinary nursing homes (NHs) and special care units for dementia (NHDs), and to evaluate the relationship between the quality of prescribing and the number of prescribers per resident, in a Swedish county. | Cross-sectional register study | Drug prescription. | Use of antipsychotics was 25.9%. Residents in NHDs, compared with NHs, had a higher prescribing rate of antipsychotics (37.5% vs 22.9%). In NHs, the proportion of younger elderly residents with antipsychotic prescriptions was 60% higher compared with older elderly residents (33% vs 20%; p<0.001). |
| Jelic V, Haglund A, Kowalski J, Langworth S, Winblad B, 2008 [46] | Donepezil treatment of severe Alzheimer’s disease in nursing home settings. A responder analysis | N=248 persons living in nursing homes.  Data were collected in 2002–2004, in Sweden.  Mean age was 84.5±6.0 in 2002, and 85.3±5.6 in 2004. | To define clinically meaningful outcomes of donepezil vs placebo treatment in severe Alzheimer’s disease and to describe characteristics of responders. | Longitudinal study with 6- month follow- up, double blinded, with a control group | Severe Impairment Battery (SIB), Alzheimer’s Disease Cooperative Study-ADL (ADCS-ADL), MMSE, Neuro-psychiatric Inventory (NPI) and Clinical Global Impression of Improvement (CGI-I). | In total 35.1% used antipsychotic drugs. Altogether 43/128 (33.5%) persons on donepezil used antipsychotics, compared with 44/120 (36.7%) controls. |
| Gustafsson M, Karlsson S, Lövheim H, 2013 [39] | Inappropriate long-term use of antipsychotic drugs is common among people with dementia living in specialized care units. | N=344 people with dementia in 40 specialized care units in nine communities, in northern Sweden.  Data were collected during 2005–2006.  Mean age at data collection was 82.1±7.8 years.  The sample was divided into two groups: people using antipsychotics (n=132) or not (n=212). | To describe antipsychotic drug therapy among people with dementia living in specialized care units in northern Sweden. | Intervention study with a 6- month follow-up | Antipsychotic drug use, ADL, cognitive function and BPSD were collected using the MDDAS. | A total of 132 (38%) people used antipsychotic drugs at the study start; 118 had been prescribed one antipsychotic drug, 13 took two, and one person had three antipsychotic drugs prescribed concomitantly.  Ninety prescriptions were for second-generation, and 57 for first-generation antipsychotics.  After 6 months, 111 of those treated with antipsychotics at baseline remained to be evaluated (seven dropouts, 14 deceased). Of these 111 people, 80 (72%) were still being treated with antipsychotics, 63 (57%) with the same dose. |
| Gustafsson M, Karlsson S, Gustafson Y, Lövheim H, 2013 [40] | Psychotropic drug use among people with dementia – a six-month follow-up study | N=353 persons with dementia at baseline, 278 of whom were followed up 6 months later in 40 specialized care units, in northern Sweden.  Data were collected during 2005–2006.  Mean age was 82 years. | To explore the long-term use of psychotropic drugs in older people with dementia. | A longitudinal study with a 6- month follow-up | Psychotropic drugs, ADL, cognitive function, and behavioural and psychological symptoms were collected using the MDDAS. | At the study start, 229 participants (82%) were prescribed at least one psychotropic drug; 111 (40%) used antipsychotics.  Sixty-two people (22%) were prescribed anxiolytics/hypnotics/sedatives and an antipsychotic drug simultaneously and 61 people (22%) used an antidepressant and an antipsychotic drug simultaneously. There were 61 persons (22%) who used three or more psychotropic drugs concomitantly.  Among the baseline users of antipsychotics, 57% still used the same dose of the same psychotropic drug after 6 months. |
| Lövheim H, Gustafson Y, Karlsson S, Sandman P-O, 2011 [47] | Comparison of behavioural and psychological symptoms of dementia and psychotropic drug treatments among old people in geriatric care in 2000 and 2007. | N=4 054 (n=2 035 in the year 2000 and  n=2 019 in 2007) persons (aged ≥65 years) with cognitive impairment living in geriatric care units, in Västerbotten, Sweden.  Data were collected in 2000 and 2007;  Mean age was 83.5±6.8 years (2000) and 84.6±6.7 years (2007). | To compare 1-week prevalence of behavioural and psychological symptoms and psychotropic drug treatment among people with cognitive impairment living in institutional care, in two large, comparable samples from 2000 and 2007. | Cross-sectional | To assess cognitive impairment and behavioural and psychological symptoms, the MDDAS was used. Cognitive impairment was assessed using Gottfries’ cognitive scale. Use of psychotropic drugs, and ADL were registered. | In 2000: use of antipsychotic drugs and anxiolytics was 26.3%,of which 14.1% were conventional and 13.4% were atypical antipsychotics.  In 2007: antipsychotic drug use (25.5%) had not changed significantly. There was a decrease in use of conventional antipsychotics (8.8%) (p*<*0.001) but an increase in atypical antipsychotics (17%) (p*<*0.001). In 2007, 66.9% of those taking antipsychotics were on atypical antipsychotics, compared with 51.0% in 2000. Use of antipsychotics, anxiolytics, hypnotics and sedatives had not changed significantly. |
| Gustafsson M, Sandman P-O, Karlsson S, Gustafson Y, Lövheim H, 2013 [48] | Association between behavioural and psychological symptoms and psychotropic drug use among old people with cognitive impairment living in geriatric care settings. | N=2 019 persons (aged ≥65 years) with cognitive impairment living in geriatric care units, in Västerbotten, Sweden.  Data were collected in 2007.  Mean age was 84.6 years. | To describe the prevalence and associated factors of psychotropic and anti-dementia drug use among old people with cognitive impairment living in geriatric care. | Cross-sectional | Psychotropic drugs, ADL, cognitive function and behavioural and psychological symptoms were collected using the MDDAS. | A total of 1 442 individuals (71%) were prescribed at least one psychotropic drug: 25% used antipsychotics (n=514), of whom 66.9% (344/514) used second-generation antipsychotics. Just over 71% (1 442/2 019) of the participants took at least one psychotropic drug. |
| Johnell K, Religa D, Eriksdotter M, 2013 [38] | Differences in drug therapy between dementia disorders in the Swedish Dementia Registry: a nationwide study of over 7,000 patients. | N=7 570 persons registered in the Swedish Dementia Registry (SveDem).  Data were collected between 2007 and 2010, in Sweden.  Mean age was 78.2±8.0 years. | To investigate whether there are differences between dementia disorders and the use of anti-dementia drugs and antipsychotics (neuroleptics) in a large population of dementia patients. | Cross-sectional | Psychotropic drugs, MMSE, age, residential settings/living conditions were collected from the register. | Overall, the use of antipsychotics was 6%; it was higher (16%) in persons with dementia with Lewy bodies (DLB) but lower (4%) in patients with Alzheimer’s disease. |
| Gustavsson M, Isaksson U, Karlsson S, Sandman P-O, Lövheim H, 2016 (unpublished at the time the search was performed) [49]) | Behavioral and psychological symptoms and psychotropic drugs among people with cognitive impairment in nursing homes  in 2007 and 2013 | N= 1971 persons from 2007 and N= 1362 persons from 2013 (aged ≥65 years) with cognitive impairment living in geriatric care units, in Västerbotten, Sweden.  Data were collected in 2007 and 2013.  Mean age was 84.7 ±6.6 years (2007), and 84.9 ±6.9 years (2013) | To analyze the prevalence of behavioral and psychological symptoms and psychotropic drug treatments among old people with cognitive impairment living in geriatric care between 2007 and 2013. | Cross-sectional at two occasions | Psychotropic drugs, ADL, cognitive function and behavioural and psychological symptoms were collected using the MDDAS. Cognitive impairment was assessed using Gottfries’ cognitive scale. | The use of antipsychotics declined from 25.4% in 2007 to 18.9 % in  2013. In those prescribed antipsychotics, 67.2% (336/500) were given atypical antipsychotics in 2007, compared to  76.3% (196/257) in 2013. The use of antidepressant drugs remained unchanged and the use of any psychotropic drug declined from 71.1% to 67.5% between the two years. |
| *The unpublished studies included* | | | | | | |
| SNAC  N=1402 |  | N=85 persons (60+) with cognitive impairment living in geriatric care units in Blekinge, Sweden. Data collected in 2000 (n=64) and 2007 (n=26). Mean age 2000 86.6+5.3 years, and 2007 84.9+7.2 years |  | Cross sectional at two occasions | Prescription of drugs from records. S-MMSE was collected in a structured interview including questionnaires | In 2000 the prevalence of antipsychotics was n=15/64, 23.4% and in 2007 the prevalence of antipsychotics was 11.5% (3/26). |
| RTPC N=292 |  | N=115 persons (65+) with cognitive impairment living in geriatric care units. In Skåne, Sweden. Data collected during 2012 and 2013. Mean age at 2012 83.6 +6.0 years and 84.1 +5.8 years at 2013. |  | A longitudinal study with a 6 month follow up | Prescription of drugs from records. S-MMSE was collected in a structured interview including questionnaires | In 2012 the prevalence of antipsychotics was 14.7% (17/115) and 12.3% (14/115) 6 months after (2013). |

ADL = Activities of Daily Living; BPSD = Behavioural and Psychological Symptoms of Dementia; MDDAS = Multi-Dimensional Dementia Assessment Scale; MMSE = Mini Mental State Examination; SIB = Severe Impairment Battery; ADCS-ADL= Alzheimer’s disease Cooperative Study-activities of daily living scale; NPI = Neuro-psychiatric Inventory; CGI-I = Clinical Global Impression of Improvement. SNAC-B= The Swedish National Study of Ageing and Care in Blekinge, RTPC= RightTimePlaceCare project.
